# Supplementary material for: MAN5, a Glycosyl Hydrolase Superfamily Protein, Is a Key Factor Involved in Cyanide-Promoted Seed Germination in Arabidopsis thaliana
Source: Genes (Basel). 2023 Jun 27;14(7):1361. doi: 10.3390/genes14071361 (PMC10379673; doi:10.3390/genes14071361)
Supplement: Supplementary file 1 [file genes-14-01361-s001.zip › genes-2416783-supplementary.pdf]

Supporting information

Table S1 Primers used for qRT-PCR

Gene_id	Primer name	Primer sequence
AT1G02310	AtMAN1-F	CGGCTGACACGGCATCT
	AtMAN1-R	TCTCCCTCCATAGTCTTCG
AT2G20680	AtMAN2-F	GATGGTAAGGCTCTGTATGT
	AtMAN2-R	GCTCGTTTATCAACTCCC
AT3G10890	AtMAN3-F	GACCATCGTCCGCACTT
	AtMAN3-R	TATTGTTTCCTCCCTCCA
AT3G10900	AtMAN4-F	AATGGCTTCAATGCTTACTG
	AtMAN4-R	CCACTTCGCCTTTACTTCT
AT4G28320	AtMAN5-F	ATGACGGTGGCTACAATG
	AtMAN5-R	TCAACTCCCAAGCGAAA
AT5G01930	AtMAN6-F	TTGGAATGACGGTAGGCA
	AtMAN6-R	CAGGCGAAGATTGTGGG
AT5G66460	AtMAN7-F	TTACAAAGATGACCCAACCA
	AtMAN7-R	GAGTGAACCGTGACGAAAT
AT2G36270	AtABI5-F	CATCCCACTAATCCTAAACC
	AtABI5-R	GCAAACACCTGCCTGAA

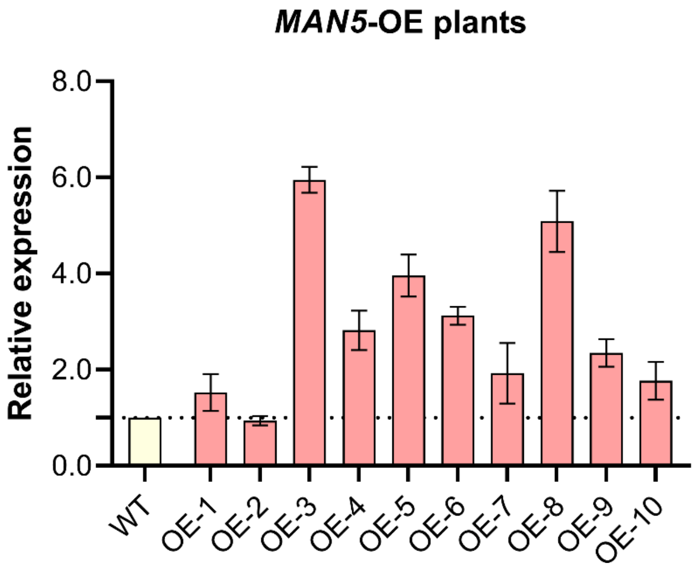

Figure S1. Relative expression of *MAN5*-OE lines. For this experiment, fresh seeds were used for gene expression analysis. The mRNA of Ten T2 generations of transgenic seeds were extracted and the cDNAs were used for qRT-PCR. In this study, OE-3 and OE-8 were selected for the whole experiments.

Table S2 Result of cis-acting element analysis of *MAN5* gene promoter

Factor or Site Name	Loc.(Str.)		Signal Sequence	SITE #
GATABOX	2	(+)	GATA	S000039
GT1CONSENSUS	2	(+)	GRWAAW	S000198
IBOXCORE	2	(+)	GATAA	S000199
ERELEE4	5	(+)	AWTTCAAA	S000037
DOFCOREZM	10	(+)	AAAG	S000265
NODCON1GM	10	(+)	AAAGAT	S000461
OSE1ROOTNODULE	10	(+)	AAAGAT	S000467
GATABOX	13	(+)	GATA	S000039
ROOTMOTIFTAPOX1	25	(-)	ATATT	S000098
ROOTMOTIFTAPOX1	26	(+)	ATATT	S000098
WBOXATNPR1	30	(+)	TTGAC	S000390
BIHD1OS	31	(-)	TGTCA	S000498
WRKY71OS	31	(+)	TGAC	S000447
ROOTMOTIFTAPOX1	37	(-)	ATATT	S000098
CURECORECR	42	(-)	GTAC	S000493
CURECORECR	42	(+)	GTAC	S000493
CAATBOX1	53	(+)	CAAT	S000028
PREATPRODH	71	(-)	ACTCAT	S000450
CACTFTPPCA1	74	(-)	YACT	S000449
EECCRCAH1	75	(-)	GANTTNC	S000494
ARR1AT	78	(-)	NGATT	S000454
CAATBOX1	88	(+)	CAAT	S000028
NODCON2GM	93	(+)	CTCTT	S000462
OSE2ROOTNODULE	93	(+)	CTCTT	S000468
ARR1AT	99	(-)	NGATT	S000454
NODCON1GM	100	(-)	AAAGAT	S000461
OSE1ROOTNODULE	100	(-)	AAAGAT	S000467
DOFCOREZM	102	(-)	AAAG	S000265
TAAAGSTKST1	102	(-)	TAAAG	S000387
GT1CONSENSUS	103	(-)	GRWAAW	S000198
IBOXCORE	104	(-)	GATAA	S000199
SREATMSD	104	(+)	TTATCC	S000470
GATABOX	105	(-)	GATA	S000039
MYBST1	105	(-)	GGATA	S000180
TATCCAYMOTIFOSRAMY3D	105	(+)	TATCCAY	S000256
TATCCAOSAMY	105	(+)	TATCCA	S000403
TATCCACHVAL21	105	(+)	TATCCAC	S000416
CAATBOX1	111	(+)	CAAT	S000028
PREATPRODH	113	(-)	ACTCAT	S000450
CACTFTPPCA1	116	(-)	YACT	S000449
EECCRCAH1	117	(-)	GANTTNC	S000494
SEF4MOTIFGM7S	125	(-)	RTTTTTR	S000103
CCA1ATLHCB1	126	(+)	AAMAATCT	S000149
ARR1AT	129	(-)	NGATT	S000454
AMYBOX1	131	(-)	TAACARA	S000020
GARE1OSREP1	131	(-)	TAACAGA	S000419
MYBCORE	132	(+)	CNGTTR	S000176

CACTFTPPCA1	137	(-)	YACT	S000449
CAATBOX1	140	(-)	CAAT	S000028
CACTFTPPCA1	145	(+)	YACT	S000449
TBOXATGAPB	146	(+)	ACTTTG	S000383
DOFCOREZM	147	(-)	AAAG	S000265
ARR1AT	150	(+)	NGATT	S000454
REALPHALGLHCB21	154	(-)	AACCAA	S000362
MYB1AT	155	(-)	WAACCA	S000408
DOFCOREZM	162	(-)	AAAG	S000265
ROOTMOTIFTAPOX1	173	(-)	ATATT	S000098
ROOTMOTIFTAPOX1	174	(+)	ATATT	S000098
GT1CONSENSUS	176	(-)	GRWAAW	S000198
IBOXCORE	177	(-)	GATAA	S000199
GATABOX	178	(-)	GATA	S000039
CAATBOX1	189	(-)	CAAT	S000028
CCAATBOX1	189	(-)	CCAAT	S000030
REALPHALGLHCB21	190	(-)	AACCAA	S000362
MYBPLANT	192	(-)	MACCWAMC	S000167
MYBPZM	192	(-)	CCWACC	S000179
BOXLCOREDCPAL	192	(-)	ACCWWCC	S000492
CACTFTPPCA1	202	(-)	YACT	S000449
EECCRCAH1	203	(-)	GANTTNC	S000494
ARR1AT	206	(-)	NGATT	S000454
BIHD1OS	211	(-)	TGTCA	S000498
WRKY71OS	211	(+)	TGAC	S000447
DOFCOREZM	225	(+)	AAAG	S000265
-10PEHVPSBD	227	(-)	TATTCT	S000392
POLASIG3	229	(+)	AATAAT	S000088
CAATBOX1	233	(-)	CAAT	S000028
CCAATBOX1	233	(-)	CCAAT	S000030
POLLEN1LELAT52	250	(-)	AGAAA	S000245
DOFCOREZM	256	(-)	AAAG	S000265
TAAAGSTKST1	256	(-)	TAAAG	S000387
GATABOX	261	(+)	GATA	S000039
CACTFTPPCA1	265	(+)	YACT	S000449
CPBCSPOR	267	(-)	TATTAG	S000491
CACTFTPPCA1	271	(+)	YACT	S000449
TATABOX4	274	(+)	TATATAA	S000111
HDZIP2ATATHB2	278	(+)	TAATMATTA	S000373
POLASIG3	279	(+)	AATAAT	S000088
POLASIG2	282	(+)	AATTAAA	S000081
ARR1AT	289	(+)	NGATT	S000454
CACTFTPPCA1	310	(-)	YACT	S000449
ANAERO2CONSENSUS	316	(-)	AGCAGC	S000478
DOFCOREZM	320	(-)	AAAG	S000265
GT1CONSENSUS	322	(-)	GRWAAW	S000198
IBOXCORE	323	(-)	GATAA	S000199
GATABOX	324	(-)	GATA	S000039
NODCON2GM	329	(+)	CTCTT	S000462
OSE2ROOTNODULE	329	(+)	CTCTT	S000468

DOFCOREZM	331	(-)	AAAG	S000265
PYRIMIDINEBOXHVEPB1	335	(+)	TTTTTTCC	S000298
GT1CONSENSUS	336	(-)	GRWAAW	S000198
GT1GMSCAM4	336	(-)	GAAAAA	S000453
GT1CONSENSUS	337	(-)	GRWAAW	S000198
-300ELEMENT	341	(-)	TGHAAARK	S000122
-300CORE	342	(-)	TGTAAAG	S000001
DOFCOREZM	342	(-)	AAAG	S000265
TAAAGSTKST1	342	(-)	TAAAG	S000387
CACTFTPPCA1	347	(+)	YACT	S000449
ARR1AT	355	(+)	NGATT	S000454
CAATBOX1	357	(-)	CAAT	S000028
ANAERO4CONSENSUS	358	(-)	GTTTHGCAA	S000480
CPBCSPOR	374	(-)	TATTAG	S000491
-300ELEMENT	384	(-)	TGHAAARK	S000122
EMHVCHORD	384	(-)	TGTAAAGT	S000452
NTBBF1ARROLB	384	(+)	ACTTTA	S000273
-300CORE	385	(-)	TGTAAAG	S000001
DOFCOREZM	385	(-)	AAAG	S000265
TAAAGSTKST1	385	(-)	TAAAG	S000387
CACTFTPPCA1	390	(+)	YACT	S000449
-300ELEMENT	391	(-)	TGHAAARK	S000122
TBOXATGAPB	391	(+)	ACTTTG	S000383
DOFCOREZM	392	(-)	AAAG	S000265
PROLAMINBOXOSGLUB1	392	(-)	TGCAAAG	S000354
CARGCW8GAT	402	(-)	CWWWWWWWWG	S000431
CARGCW8GAT	402	(+)	CWWWWWWWWG	S000431
ROOTMOTIFTAPOX1	419	(-)	ATATT	S000098
GATABOX	421	(-)	GATA	S000039
MYBST1	421	(-)	GGATA	S000180
TATCCAOSAMY	421	(+)	TATCCA	S000403
CACTFTPPCA1	438	(+)	YACT	S000449
CURECORECR	442	(-)	GTAC	S000493
CURECORECR	442	(+)	GTAC	S000493
CAATBOX1	446	(-)	CAAT	S000028
CACTFTPPCA1	456	(-)	YACT	S000449
POLASIG3	459	(-)	AATAAT	S000088
WBOXHVISO1	469	(+)	TGACT	S000442
WRKY71OS	469	(+)	TGAC	S000447
WBOXNTERF3	469	(+)	TGACY	S000457
NODCON2GM	472	(+)	CTCTT	S000462
OSE2ROOTNODULE	472	(+)	CTCTT	S000468
DOFCOREZM	474	(-)	AAAG	S000265
MYBCORE	484	(-)	CNGTTR	S000176
MYB2AT	484	(+)	TAACTG	S000177
MYB2CONSENSUSAT	484	(+)	YAACKG	S000409
MYBCORE	487	(+)	CNGTTR	S000176
POLASIG1	492	(+)	AATAAA	S000080
DOFCOREZM	496	(+)	AAAG	S000265
WBOXHVISO1	501	(-)	TGACT	S000442

WBOXNTERF3	501	(-)	TGACY	S000457
WRKY71OS	502	(-)	TGAC	S000447
GTGANTG10	503	(-)	GTGA	S000378
LTREATLTI78	505	(+)	ACCGACA	S000157
DRE2COREZMRAB17	505	(+)	ACCGAC	S000402
DRECRTCOREAT	505	(+)	RCCGAC	S000418
CBFHV	505	(+)	RYCGAC	S000497
LTRECOREATCOR15	506	(+)	CCGAC	S000153
TBOXATGAPB	517	(-)	ACTTTG	S000383
DOFCOREZM	518	(+)	AAAG	S000265
CACTFTPPCA1	520	(-)	YACT	S000449
CACTFTPPCA1	527	(+)	YACT	S000449
BOXIINTPATPB	537	(-)	ATAGAA	S000296
MARABOX1	542	(-)	AATAAAYAAA	S000063
POLASIG1	542	(-)	AATAAA	S000080
TATABOX5	543	(+)	TTATTT	S000203
POLASIG1	546	(-)	AATAAA	S000080
POLASIG3	549	(-)	AATAAT	S000088
CAATBOX1	552	(-)	CAAT	S000028
GT1CONSENSUS	558	(-)	GRWAAW	S000198
DPBFCOREDCDC3	566	(+)	ACACNNG	S000292
POLLEN1LELAT52	581	(-)	AGAAA	S000245
CRTDREHVCBF2	589	(-)	GTCGAC	S000411
CBFHV	589	(-)	RYCGAC	S000497
CRTDREHVCBF2	589	(+)	GTCGAC	S000411
CBFHV	589	(+)	RYCGAC	S000497
POLLEN1LELAT52	597	(+)	AGAAA	S000245
GT1CONSENSUS	598	(+)	GRWAAW	S000198
GATABOX	605	(+)	GATA	S000039
CACTFTPPCA1	608	(-)	YACT	S000449
CURECORECR	609	(-)	GTAC	S000493
CURECORECR	609	(+)	GTAC	S000493
CACTFTPPCA1	610	(+)	YACT	S000449
MYBCORE	612	(+)	CNGTTR	S000176
ROOTMOTIFTAPOX1	622	(+)	ATATT	S000098
-10PEHVPSBD	623	(+)	TATTCT	S000392
AMYBOX1	636	(+)	TAACARA	S000020
MYBGAHV	636	(+)	TAACAAA	S000181
GAREAT	636	(+)	TAACAAR	S000439
POLLEN1LELAT52	648	(+)	AGAAA	S000245
GT1CONSENSUS	649	(+)	GRWAAW	S000198
GT1GMSCAM4	649	(+)	GAAAAA	S000453
DOFCOREZM	659	(+)	AAAG	S000265
SURECOREATSULTR11	665	(+)	GAGAC	S000499
ANAERO3CONSENSUS	674	(-)	TCATCAC	S000479
GTGANTG10	674	(+)	GTGA	S000378
GTGANTG10	687	(-)	GTGA	S000378
CARGCW8GAT	690	(-)	CWWWWWWWWG	S000431
CARGCW8GAT	690	(+)	CWWWWWWWWG	S000431
CAATBOX1	701	(-)	CAAT	S000028

TATABOX2	707	(-)	TATAAAT	S000109
CAATBOX1	714	(+)	CAAT	S000028
WBBOXPCWRKY1	718	(+)	TTTGACY	S000310
WBOXATNPR1	719	(+)	TTGAC	S000390
WBOXHVISO1	720	(+)	TGACT	S000442
WRKY71OS	720	(+)	TGAC	S000447
WBOXNTERF3	720	(+)	TGACY	S000457
RAV1AAT	728	(+)	CAACA	S000314
SREATMSD	734	(-)	TTATCC	S000470
MYBST1	734	(+)	GGATA	S000180
GATABOX	735	(+)	GATA	S000039
GT1CONSENSUS	735	(+)	GRWAAW	S000198
IBOXCORE	735	(+)	GATAA	S000199
DOFCOREZM	740	(+)	AAAG	S000265
ARR1AT	744	(-)	NGATT	S000454
SV40COREENHAN	747	(-)	GTGGWWHG	S000123
EBOXBNNAPA	754	(-)	CANNTG	S000144
MYCCONSENSUSAT	754	(-)	CANNTG	S000407
EBOXBNNAPA	754	(+)	CANNTG	S000144
MYCCONSENSUSAT	754	(+)	CANNTG	S000407
CAATBOX1	762	(-)	CAAT	S000028
BIHD1OS	764	(+)	TGTCA	S000498
WBOXATNPR1	765	(-)	TTGAC	S000390
WRKY71OS	765	(-)	TGAC	S000447
CIACADIANLELHC	767	(+)	CAANNNNATC	S000252
GATABOX	773	(-)	GATA	S000039
NODCON2GM	781	(+)	CTCTT	S000462
OSE2ROOTNODULE	781	(+)	CTCTT	S000468
DOFCOREZM	783	(-)	AAAG	S000265
GT1CONSENSUS	785	(-)	GRWAAW	S000198
IBOXCORE	786	(-)	GATAA	S000199
GATABOX	787	(-)	GATA	S000039
BP5OSWX	799	(+)	CAACGTG	S000436
T/GBOXATPIN2	800	(+)	AACGTG	S000458
ABRERATCAL	800	(+)	MACGYGB	S000507
ACGTATERD1	801	(-)	ACGT	S000415
ABRELATERD1	801	(+)	ACGTG	S000414
ACGTATERD1	801	(+)	ACGT	S000415
DOFCOREZM	818	(+)	AAAG	S000265
CACTFTPPCA1	820	(-)	YACT	S000449
GTGANTG10	821	(+)	GTGA	S000378
ARR1AT	822	(+)	NGATT	S000454
EECCRCAH1	823	(+)	GANTTNC	S000494
GT1CONSENSUS	824	(-)	GRWAAW	S000198
NODCON2GM	830	(-)	CTCTT	S000462
OSE2ROOTNODULE	830	(-)	CTCTT	S000468
XYLAT	836	(+)	ACAAAGAA	S000510
DOFCOREZM	838	(+)	AAAG	S000265
POLLEN1LELAT52	840	(+)	AGAAA	S000245
TATABOX5	842	(-)	TTATTT	S000203

SEF1MOTIF	843	(-)	ATATTTAWW	S000006
POLASIG1	843	(+)	AATAAA	S000080
ROOTMOTIFTAPOX1	847	(-)	ATATT	S000098
CACTFTPPCA1	854	(+)	YACT	S000449
ROOTMOTIFTAPOX1	859	(-)	ATATT	S000098
RAV1AAT	863	(-)	CAACA	S000314
ROOTMOTIFTAPOX1	885	(-)	ATATT	S000098
ROOTMOTIFTAPOX1	886	(+)	ATATT	S000098
GT1CONSENSUS	888	(-)	GRWAAW	S000198
POLLEN1LELAT52	890	(-)	AGAAA	S000245
BOXIINTPATPB	891	(-)	ATAGAA	S000296
L1BOXATPDF1	898	(+)	TAAATGYA	S000386
POLASIG3	906	(-)	AATAAT	S000088
TATABOX5	907	(+)	TTATTT	S000203
ARR1AT	912	(+)	NGATT	S000454
ANAERO1CONSENSUS	915	(-)	AAACAAA	S000477
GT1CORE	935	(-)	GGTTAA	S000125
DOFCOREZM	940	(-)	AAAG	S000265
PREATPRODH	955	(-)	ACTCAT	S000450
CAREOSREP1	957	(-)	CAACTC	S000421
SITEIIATCYTC	961	(+)	TGGGCY	S000474
GTGANTG10	966	(-)	GTGA	S000378
CAATBOX1	969	(+)	CAAT	S000028
GTGANTG10	973	(-)	GTGA	S000378
GTGANTG10	978	(-)	GTGA	S000378
EBOXBNNAPA	979	(-)	CANNTG	S000144
MYCCONSENSUSAT	979	(-)	CANNTG	S000407
MYCATERD1	979	(-)	CATGTG	S000413
EBOXBNNAPA	979	(+)	CANNTG	S000144
MYCATRD22	979	(+)	CACATG	S000174
MYCCONSENSUSAT	979	(+)	CANNTG	S000407
ARR1AT	983	(+)	NGATT	S000454
CAATBOX1	985	(-)	CAAT	S000028
CAATBOX1	991	(+)	CAAT	S000028
GT1CONSENSUS	1001	(-)	GRWAAW	S000198
GT1GMSCAM4	1001	(-)	GAAAAA	S000453
POLLEN1LELAT52	1003	(-)	AGAAA	S000245
ROOTMOTIFTAPOX1	1010	(-)	ATATT	S000098
GATABOX	1012	(-)	GATA	S000039
BIHD1OS	1025	(-)	TGTCA	S000498
WRKY71OS	1025	(+)	TGAC	S000447
CACTFTPPCA1	1028	(+)	YACT	S000449
EBOXBNNAPA	1038	(-)	CANNTG	S000144
MYCATRD22	1038	(-)	CACATG	S000174
MYCCONSENSUSAT	1038	(-)	CANNTG	S000407
EBOXBNNAPA	1038	(+)	CANNTG	S000144
MYCCONSENSUSAT	1038	(+)	CANNTG	S000407
MYCATERD1	1038	(+)	CATGTG	S000413
SORLIP1AT	1041	(-)	GCCAC	S000482
SORLIP2AT	1043	(-)	GGGCC	S000483

POLASIG1	1050	(-)	AATAAA	S000080
MARTBOX	1051	(+)	TTWTWTTWTT	S000067
TATABOX5	1051	(+)	TTATTT	S000203
PYRIMIDINEBOXHVEPB1	1056	(+)	TTTTTTCC	S000298
GT1CONSENSUS	1057	(-)	GRWAAW	S000198
GT1GMSCAM4	1057	(-)	GAAAAA	S000453
GT1CONSENSUS	1058	(-)	GRWAAW	S000198
NODCON2GM	1065	(-)	CTCTT	S000462
OSE2ROOTNODULE	1065	(-)	CTCTT	S000468
ARR1AT	1068	(+)	NGATT	S000454
EBOXBNNAPA	1073	(-)	CANNTG	S000144
MYCCONSENSUSAT	1073	(-)	CANNTG	S000407
EBOXBNNAPA	1073	(+)	CANNTG	S000144
MYCCONSENSUSAT	1073	(+)	CANNTG	S000407
PYRIMIDINEBOXHVEPB1	1083	(+)	TTTTTTCC	S000298
GT1CONSENSUS	1084	(-)	GRWAAW	S000198
GT1GMSCAM4	1084	(-)	GAAAAA	S000453
GT1CONSENSUS	1085	(-)	GRWAAW	S000198
GTGANTG10	1092	(+)	GTGA	S000378
GATABOX	1094	(+)	GATA	S000039
CACTFTPPCA1	1096	(+)	YACT	S000449
PREATPRODH	1097	(+)	ACTCAT	S000450
POLASIG2	1106	(+)	AATTAAA	S000081
DOFCOREZM	1119	(-)	AAAG	S000265
NTBBF1ARROLB	1123	(-)	ACTTTA	S000273
TAAAGSTKST1	1123	(+)	TAAAG	S000387
DOFCOREZM	1124	(+)	AAAG	S000265
RAV1AAT	1132	(+)	CAACA	S000314
RAV1AAT	1135	(+)	CAACA	S000314
ROOTMOTIFTAPOX1	1139	(+)	ATATT	S000098
CPBCSPOR	1140	(+)	TATTAG	S000491
SURECOREATSULTR11	1145	(-)	GAGAC	S000499
NODCON2GM	1147	(+)	CTCTT	S000462
OSE2ROOTNODULE	1147	(+)	CTCTT	S000468
DOFCOREZM	1149	(-)	AAAG	S000265
ARR1AT	1161	(-)	NGATT	S000454
GT1CORE	1174	(+)	GGTTAA	S000125
DOFCOREZM	1188	(-)	AAAG	S000265
GT1CONSENSUS	1189	(-)	GRWAAW	S000198
GT1GMSCAM4	1189	(-)	GAAAAA	S000453
GT1CONSENSUS	1190	(-)	GRWAAW	S000198
NODCON2GM	1195	(+)	CTCTT	S000462
OSE2ROOTNODULE	1195	(+)	CTCTT	S000468
DOFCOREZM	1200	(-)	AAAG	S000265
ELRECOREPCRP1	1208	(-)	TTGACC	S000142
WBOXNTERF3	1208	(-)	TGACY	S000457
WBOXATNPR1	1209	(-)	TTGAC	S000390
WRKY71OS	1209	(-)	TGAC	S000447
DOFCOREZM	1222	(-)	AAAG	S000265
MARTBOX	1238	(+)	TTWTWTTWTT	S000067

POLASIG1	1242	(-)	AATAAA	S000080
TATABOX5	1243	(+)	TTATTT	S000203
TATABOX2	1245	(-)	TATAAAT	S000109
SEF1MOTIF	1248	(-)	ATATTTAWW	S000006
TATABOX2	1248	(+)	TATAAAT	S000109
ROOTMOTIFTAPOX1	1252	(-)	ATATT	S000098
GATABOX	1254	(-)	GATA	S000039
MYBST1	1254	(-)	GGATA	S000180
TATCCAOSAMY	1254	(+)	TATCCA	S000403
CCAATBOX1	1257	(+)	CCAAT	S000030
CAATBOX1	1258	(+)	CAAT	S000028
SEF4MOTIFGM7S	1260	(+)	RTTTTTR	S000103
EVENINGAT	1270	(-)	AAAATATCT	S000385
GATABOX	1271	(+)	GATA	S000039
LECPLEACS2	1272	(-)	TAAAATAT	S000465
ROOTMOTIFTAPOX1	1272	(+)	ATATT	S000098
CACTFTPPCA1	1282	(-)	YACT	S000449
CACTFTPPCA1	1305	(-)	YACT	S000449
-300ELEMENT	1307	(+)	TGHAAARK	S000122
ARR1AT	1312	(-)	NGATT	S000454
DPBFCOREDCDC3	1320	(-)	ACACNNG	S000292
MARTBOX	1329	(+)	TTWTWTTWTT	S000067
MARTBOX	1330	(+)	TTWTWTTWTT	S000067
MARTBOX	1331	(+)	TTWTWTTWTT	S000067
MARTBOX	1332	(+)	TTWTWTTWTT	S000067
GTGANTG10	1348	(-)	GTGA	S000378
PYRIMIDINEBOXOSRAMY1A	1365	(+)	CCTTTT	S000259
-300ELEMENT	1366	(-)	TGHAAARK	S000122
DOFCOREZM	1366	(-)	AAAG	S000265
GT1CONSENSUS	1375	(+)	GRWAAW	S000198
PYRIMIDINEBOXOSRAMY1A	1385	(-)	CCTTTT	S000259
DOFCOREZM	1386	(+)	AAAG	S000265
GT1CONSENSUS	1404	(-)	GRWAAW	S000198
POLLEN1LELAT52	1406	(-)	AGAAA	S000245
DOFCOREZM	1412	(-)	AAAG	S000265
GTGANTG10	1415	(-)	GTGA	S000378
DOFCOREZM	1420	(+)	AAAG	S000265
POLLEN1LELAT52	1422	(+)	AGAAA	S000245
GT1CONSENSUS	1423	(+)	GRWAAW	S000198
TATABOX3	1433	(-)	TATTAAT	S000110
SURE1STPAT21	1436	(+)	AATAGAAAA	S000186
BOXIINTPATPB	1437	(+)	ATAGAA	S000296
POLLEN1LELAT52	1439	(+)	AGAAA	S000245
GT1CONSENSUS	1440	(+)	GRWAAW	S000198
GT1GMSCAM4	1440	(+)	GAAAAA	S000453
ROOTMOTIFTAPOX1	1444	(-)	ATATT	S000098
ROOTMOTIFTAPOX1	1445	(+)	ATATT	S000098
POLASIG3	1447	(-)	AATAAT	S000088

MARTBOX	1448	(+)	TTWTWTTWTT	S000067
TATABOX5	1448	(+)	TTATTT	S000203
TATABOX3	1466	(-)	TATTAAT	S000110
SURE1STPAT21	1469	(+)	AATAGAAAA	S000186
BOXIINTPATPB	1470	(+)	ATAGAA	S000296
POLLEN1LELAT52	1472	(+)	AGAAA	S000245
GT1CONSENSUS	1473	(+)	GRWAAW	S000198
GT1GMSCAM4	1473	(+)	GAAAAA	S000453
ROOTMOTIFTAPOX1	1477	(-)	ATATT	S000098
CACTFTPPCA1	1482	(-)	YACT	S000449
DOFCOREZM	1495	(-)	AAAG	S000265
TAAAGSTKST1	1495	(-)	TAAAG	S000387
